# Cumulative Metabolic and Epigenetic Effects of Paternal and/or Maternal Supplementation with Arachidonic Acid across Three Consecutive Generations in Mice

**DOI:** 10.3390/cells11061057

**Published:** 2022-03-21

**Authors:** Carmen de la Rocha, Dalia Rodríguez-Ríos, Enrique Ramírez-Chávez, Jorge Molina-Torres, José de Jesús Flores-Sierra, Luis M. Orozco-Castellanos, Juan P. Galván-Chía, Atenea Vázquez Sánchez, Silvio Zaina, Gertrud Lund

**Affiliations:** 1 Department of Genetic Engineering, CINVESTAV Irapuato Unit, Irapuato 36500, Mexico; carmen_de_la_rocha@hotmail.com (C.d.l.R.); dalia.rodriguez@cinvestav.mx (D.R.-R.); atenea.vazquez@cinvestav.mx (A.V.S.); 2 Department of Biotechnology and Biochemistry, CINVESTAV Irapuato Unit, Irapuato 36500, Mexico; enrique.ramirez@cinvestav.mx (E.R.-C.); jmolinat@cinvestav.mx (J.M.-T.); 3 Department of Medical Sciences, Division of Health Sciences, León Campus, University of Guanajuato, León 37000, Mexico; jesus.fs@purisima.tecnm.mx (J.d.J.F.-S.); szaina@ugto.mx (S.Z.); 4 Department of Pharmacology, Division of Natural and Exact Sciences, Guanajuato Campus, University of Guanajuato, Guanajuato 36000, Mexico; orozcoz@hotmail.com (L.M.O.-C.); jgalvanchia@gmail.com (J.P.G.-C.)

**Keywords:** arachidonic acid, DNA methylation, cis-7-hexadecenoic acid, stearoyl-CoA desaturase 1, growth, transgenerational, cumulative

## Abstract

Apart from the known associations between arachidonic acid (AA), weight gain, and neurological and immune function, AA exposure leads to alterations in global and gene-specific DNA methylation (DNAm) and fatty acid (FA) content in human cultured cells. However, it is unknown as to whether the latter effects occur in vivo and are maintained over extended periods of time and across generations. To address this issue, we asked whether AA supplementation for three consecutive generations (prior to coitus in sires or in utero in dams) affected offspring growth phenotypes, in addition to liver DNAm and FA profiles in mice. Twelve-week-old BALB/c mice were exposed daily to AA dissolved in soybean oil (vehicle, VH), or VH only, for 10 days prior to mating or during the entire pregnancy (20 days). On average, 15 mice were supplemented per generation, followed by analysis of offspring body weight and liver traits (x average = 36 and 10 per generation, respectively). Body weight cumulatively increased in F2 and F3 offspring generations and positively correlated with milligrams of paternal or maternal offspring AA exposure. A concomitant increase in liver weight was observed. Notably, akin to AA-challenged cultured cells, global DNAm and cis-7-hexadecenoic acid (16:1n-9), an anti-inflammatory FA that is dependent on stearoyl-CoA desaturase 1 (SCD1) activity, increased with milligrams of AA exposure. In accordance, liver *Scd1* promoter methylation decreased with milligrams of germline AA exposure and was negatively correlated with liver weight. Our results show that mice retain cellular memories of AA exposure across generations that could potentially be beneficial to the innate immune system.

## 1. Introduction

A retrospective epidemiological study published by Barker and Osmond in 1986 showed that death from cardiovascular disease in adulthood was negatively correlated with birth weight [1]. That study led to the concept of “Developmental Origins of Health and Disease” (DOHaD) and fueled investigations demonstrating that factors such as nutrition, stress, infections, and exposure to toxins in early development impact cellular phenotypes, not only within, but also across generations [2]. 

Regarding underlying molecular mechanisms of DOHaD, DNA methylation (DNAm) has received the most attention, largely due to its essential role in normal development, in addition to the extensive reprogramming it undergoes during gametogenesis and fetal development [3]. The first identified link between DNAm and DOHaD was the increase in mouse agouti promoter DNAm in offspring of dams exposed to a methyl-donor rich diet prior to and during pregnancy and lactation [4]. A further demonstration of the potential importance of DNAm in DOHaD is the dysregulation of genomic imprinting associated with maternal or paternal poor nutrition and obesity [5]. Additionally, paternal exposure to specific environmental factors results in transgenerational transmission of specific phenotypes such infertility, with the participation of imprinted genes [6,7]. Subsequent additional dietary intervention studies have confirmed similar associations, not only to gene-specific DNAm, but also to alterations in specific histone modifications and non-coding RNAs [8,9,10,11,12,13,14,15,16,17,18,19]. Taken together, the above evidence justifies a careful survey of both maternal and paternal effects.

Most DOHaD transgenerational studies have focused on epigenetic effects of dietary protein, micronutrients, or fat composition [20,21]. With respect to the latter, interventions have mainly focused on increasing total fat content, altering n-3/n-6 fatty acid (FA) ratios, or using oils enriched in specific FA, while only few studies have analyzed the effects of individual FA [22,23]. The latter is important given that the degree of FA saturation differentially affects both global and gene-specific DNAm profiles [24,25,26,27,28,29,30,31]. For example, a 24 h stimulation with arachidonic acid (C20:4n-6, AA) induces a dose-dependent increase in global DNAm, while oleic acid shows the opposite tendency in THP-1 monocytic cells [28]. In that model, the AA-induced increase in global DNAm is entirely dependent on peroxisome proliferator-activated receptor alpha (PPARα) activity and mitochondrial beta-oxidation [28]. In addition, inhibition of histone deacetylase SIRTUIN 1 (SIRT1) activity affects global DNAm [28], presumably via deacetylation and activation of PPARγ coactivator 1 α (PPARGC1A) [32], a known coactivator of PPARα [33]. Moreover, THP-1 cells respond acutely to AA by the formation of lipid droplets, enriched in saturated and monounsaturated fatty acids (SFA and MUFA, respectively) [34,35]. Of particular interest is the accumulation of the anti-inflammatory cis-7-hexadecenoic (HDA) MUFA. HDA is a positional isomer of palmitoleic acid that is generated by stearoyl-coenzyme A desaturase 1 (SCD1)-mediated desaturation of stearic acid to oleic acid and one round of beta-oxidation [35]. 

AA, in addition to docosahexaenoic acid (DHA, 22:6n-3), have historically been added to infant formula to mimic FA breast milk concentrations, i.e., equal or greater amounts of AA relative to DHA [36]. Furthermore, both FA play essential roles in infant development, particularly in brain and immune development [37]. However, the new infant formula recommendations of the European Union no longer require the inclusion of AA, notwithstanding that only few studies have analyzed the effects of an AA supplement independently of the presence of DHA [38]. Furthermore, the impact of early AA exposure on later development, both within and across generations, is unknown. The question is relevant from the viewpoint of human nutrition in general, as a dietary survey of the North American population concluded that AA is present at levels that are comparable to other PUFA (2014 Position of the Academy of Nutrition and Dietetics: Dietary Fatty Acids for Healthy Adults, University of Nebraska, in: https://digitalcommons.unl.edu/nutritionfacpub. Accessed on 21 February 2022). Moreover, evidence exists that the contribution of AA to the Western diet has been underestimated [39]. Oppositely, dietary intake of AA in low-income countries is below than current recommended levels [40]. Several outcomes can be envisaged, including ones in which AA lacks any effect across generations, acts only on the initially exposed generations, or exerts cumulative effects. Based on the above evidence, our objective was to probe for effects of AA supplementation across three consecutive generations on offspring growth, FA content, and DNAm in mice. Our data showed that AA supplementation leads to a cumulative increase in body weight across generations, accompanied by alterations in DNAm and FA content, akin to those previously described following stimulation of THP-1 cell with AA.

## 2. Materials and Methods

### 2.1. Animal Experiments

Animal experiments were performed according to Mexican NOM-062-ZOO-1999 guidelines. The described mouse protocol was approved by the Ethical Committee of the Department of Medical Sciences, University of Guanajuato. BALB/c mice were housed under standard experimental conditions with ad libitum access to water and a standard laboratory diet (Laboratory Rodent diet 5001, Purina, St. Louis, MO, USA). In the diet, AA represented 0.18% of total FA (Table S1).

### 2.2. Maternal and Paternal Supplementation Experiments

Twelve-week-old BALB/c sires or dams received a daily supplement of 98.5% pure AA (Sigma-Aldrich, St. Louis, MO, USA, A3611). The AA dose represented 0.45% of total FA (or 0.05% of total daily energy) and was within values recommended for infant nutrition [41]. Specifically, sires and dams received a supplement of 1.35 and 1.1 mg (≈37 mg/kg/day) corresponding to 1.2 and 1.0 μL of AA, respectively, according to an AA density of 0.922 g/mL [42]. In either case, the final volume administered was adjusted to 5 μL by the addition of a vehicle (VH) oil (Nutrioli^®^, a commercial soybean oil) devoid of AA (Table S1). Control mice received 5 μL VH only. To minimize oxidation, upon vial opening, AA was immediately dissolved in VH, which contained 0.01% of the antioxidant tert-butylhydroquinone, and the 5 μL aliquots were stored at −20 °C until use. The 5 μL AA+VH (AA) or control (VH only) supplements were administrated orally with a blunted pipette tip to avoid mouse injury. Both supplements were very palatable to mice and therefore were completely ingested. Dams received supplements during the entire pregnancy, i.e., for 20 consecutive days following the appearance of a vaginal plug. In sires, supplements were administered for a period of 10 days prior to mating. In either case, mice were randomly allocated to each experimental group and AA and VH supplements were administered within similar time frames at each consecutive generation. Females were housed in groups of 2–3 individuals per cage, while male mice were housed individually. Food intake was only assayed of paternally exposed mice. For each pregnant dam we also recorded litter size and male/female ratio of offspring, in addition to weight at day 1 of pregnancy (i.e., supplemental day 1).

### 2.3. Analysis of Offspring of AA and VH Supplemented Dams and Sires

Offspring of supplemented mice was analyzed at weaning (day 28 post-partum) as follows at each generation: (1) all offspring was weighed and sexed; (2) an average of 5 male and female mice were randomly selected from litters of different dams and euthanized by cervical dislocation; liver, brain, testes, intestine, uterus, and ovary were dissected, weighed, and stored in RNAlater (Ambion, Austin, TX, USA) at −20 °C; (3) The remaining offspring were maintained on a standard diet until postnatal week 12 where female or male F1 and F2 offspring were re-exposed to AA and VH supplements as described above.

Global and gene specific DNAm, individual FA, and total fat was analyzed of liver tissue pooled from a total of six mice per generation (three males and three females). Individual FA were determined as fatty acid methyl esters (FAMES) using 20 mg of liver tissue as previously described [26]. The isomers of C18:1 and C16:1 were identified using commercial standards of elaidic acid, C16:1n-9 and C16:1n-7 (Sigma-Aldrich, St. Louis, MO, USA no. E4637, and Cayman Chemical, Ann Arbor, MO, USA no. 154-10009871 and 154-10007290, respectively). Total liver fat was determined as percentage weight loss following extraction of ≈30 mg of liver tissue in 10 mL of chloroform for 3 weeks at 4 °C. Global and gene specific DNAm was performed as follows: DNA was quantified using SYBR Green (Sigma-Aldrich, St. Louis, MO, USA), and 50 ng was used for duplicate measurements of global DNAm with MethylFlash™ (Methylated DNA Quantification Kit, Colorimetric, Epigentek, Farmingdale, NY, USA). The same samples were subjected to gene-specific promoter methylation analysis by pyrosequencing following bisulfite treatment (EpigenDx Inc., Worcester, MA, USA). The following promoter regions were analyzed: *Ppargc1a*: chr5: 51945395-51945241; *Scd1*: chr19: 44408468-44408458; and *Fads2*: chr19: 1010826-10101788.

### 2.4. RT-PCR Analysis

Total RNA was extracted using TRIzol™ Reagent (Invitrogen™, Waltham, MA, USA). Following treatment with DNase I, Amplification Grade (Invitrogen™, Waltham, MA, USA), cDNA was synthesized from 1 μg of RNA using SuperScript™ III First-Strand Synthesis System (Invitrogen™, Waltham, MA, USA). A 1/50 vol. was used for semiquantitative PCR with the following forward and reverse primer pairs: *Scd1*, 5′-CACCTGCCTCTTCGGGATTT-3′ and 5′-CTTTGACAGCCGGGTGTTTG-3′, respectively; *Ppargc1a*, 5′-CAGGAACAGCAGCAGAGACA-3′ and 5′-ATGGTCACCAAACAGCCGAA-3′, respectively. RT-PCR products were quantitated by densitometric scanning (Quantity One, Bio-Rad, Hercules, CA, USA), and data were normalized to *M. musculus* ribosomal protein 36b4 (forward primer: 5′-CACCTGCCTCTTCGGGATTT-3′; reverse primer: 5′-CTTTGACAGCCGGGTGTTTG-3′).

### 2.5. Statistical Analysis

ANOVA with Scheffé’s post hoc test or paired *t*-tests were used for comparisons between generations and across supplemented groups. Pearson’s test was used for correlations across generations. All tests were performed with SPSS 15.0 software.

## 3. Results

### 3.1. Maternal and Paternal Supplementation Experiments

**Figure 1:**
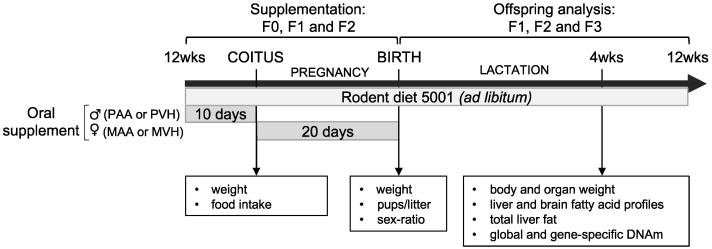
Mouse supplementation experiments. Three-month-old mice were supplemented with AA dissolved in vehicle (VH; soybean oil), or VH only, maternally (MAA and MVH) or paternally (PAA and PVH, respectively) for three consecutive generations (F0, F1, and F2). Administration of AA and VH supplements were performed within similar time frames. The effect of either FA supplement was subsequently analyzed in one-month-old F1, F2, and F3 offspring of AA- or VH-exposed dams or sires.

Dietary interventions and analyses were aimed at understanding how paternal or maternal AA (PAA or MAA, respectively) or VH (PVH or MVH, respectively) supplementation for three consecutive generations affected offspring weight (Figure 1).

**Figure 2:**
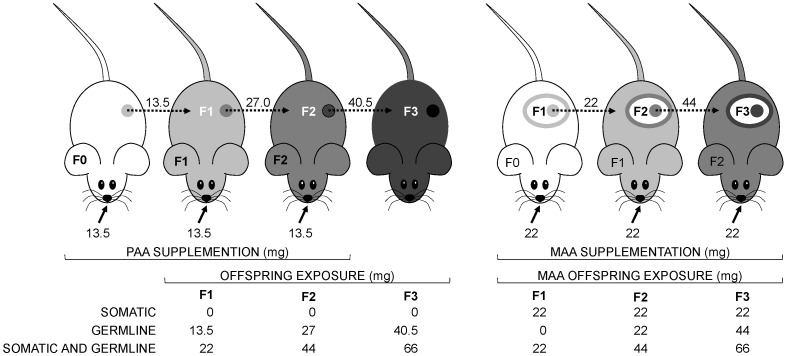
Cumulative germline and/or somatic offspring AA exposure of paternally and maternally supplemented mice. Filled arrows indicate total mg of AA supplement (calculated by multiplying mg of daily supplement by days of exposure) administered at each generation (F0, F1, and F2); dotted arrows indicate milligrams of germline or soma AA exposure (circles and ovals, respectively) of F1, F2 and F3 offspring generations. Cumulative AA exposure somatic and/or germline offspring exposure at each generation are indicated below each mouse generation. Similar calculations were performed of paternal and maternal vehicle supplements.

Briefly, PAA and PVH supplements were administered for 10 days prior to crossing with non-supplemented females, while MAA and MVH supplementation was for the entire 20 day pregnancy period. Paternal supplementation experiments were realized between August 2014 and August 2015, while the corresponding maternal experiments were conducted between November 2014 and August 2015. We supplemented an average of 15 sires or dams per supplement per generation and recorded body weight of an average of 36 offspring per supplement per generation (Table S2). This experimental design allowed us to probe for cumulative effects of germline or somatic AA exposure or of the two exposures compounded on PAA and MAA offspring body weight, respectively (Figure 2).

### 3.2. Effects of Offspring AA and VH Exposure on Body Weight

First, we detailed the effects of paternal or maternal AA and control (VH) supplements on body weight of the supplemented F0 generation, i.e., the weight of three-month-old sires or dams that were orally supplemented for 10 or 20 days, respectively. Neither baseline nor relative weight gain, differed significantly across AA- and VH-supplemented mice (Figure S1A,B). Furthermore, as in previously published studies of mouse and rat models [43,44,45], AA supplementation did not affect food intake (Figure S1C).

**Figure 3:**
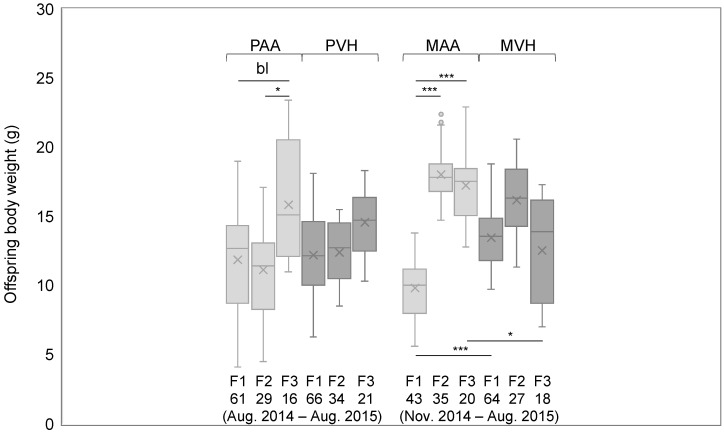
Offspring body weight of mice supplemented paternally or maternally for three consecutive generations. Horizontal lines indicate significant differences between each experimental group; ^bl^ *p* < 0.1, * *p* < 0.05, *** *p* < 0.001; supplemental period and n of each group is indicated under each bar graph; PAA and MAA: paternal and maternal AA supplementation, respectively; PVH and MVH: paternal and maternal VH supplementation, respectively.

Since we were interested in generational effects of germline or in utero AA supplementation, we first asked if offspring body weight differed across the three consecutive supplemented generations (Figure 3). 

We observed a significant and borderline significant increase in body weight of F3 relative to F2 or F1 PAA offspring (*p* = 0.0248 and *p* = 0.0596, respectively). Likewise, MAA offspring showed significant weight gain in F3 and F2 generations relative to the F1 generation (*p* = 6.18 × 10^−11^ and *p* = 5.42 × 10^−20^, respectively). Conversely, offspring of PVH- and MVH-supplemented mice showed no significant increase or decrease in body weight across generations (Figure 3). Notably, the described AA and VH supplementation weight phenotypes were observed in both female and male offspring (Figure S2). Comparing MAA and MVH offspring generations, F1 and F3 weight was significantly decreased and increased, respectively, of AA relative to VH offspring (*p* = 0.0003 and *p* = 0.0361, respectively) (Figure 3). Taken together, the data suggest that PAA and MAA exposure for three generations leads to a cumulative body weight gain. In the case of MAA offspring, the initial response to prior AA exposure is weight loss, followed by sustained weight gain for two consecutive generations. Furthermore, the fact that offspring of AA- and VH-exposed mice were bred at almost similar time periods rules out any seasonal effect.

**Figure 4:**
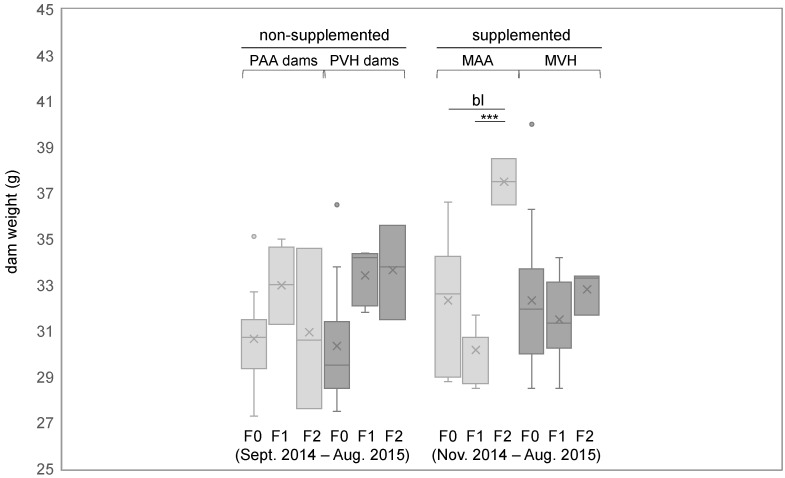
Body weight of three-month-old dams at supplemental day 1. Y-axis indicates dam body weight at supplemental day 1: day 1 of pregnancy. PAA and PVH dams: non-supplemented dams that were crossed to AA- or VH-supplemented sires. ^bl^ *p* < 0.1, *** *p* < 0.001. Other symbols are as in Figure 3.

Next, we asked if the cumulative generational weight gain in offspring of AA-supplemented dams was conserved later in adults. To that end, we compared body weight of three-month-old dams of each generation at supplemental day 1 (day 1 of pregnancy). We compared both AA and VH supplemented dams, in addition to non-supplemented dams, i.e., the dams that were bred with AA- or VH-supplemented sires (PAA and PVH dams, respectively). The body weights of each experimental group are shown in Table S3. Only AA—but neither VH- nor PAA/PVH-supplemented dams—showed a significant (or borderline significant) increase in body weight in F2 compared to F1 or F0 generations (*p* = 0.0008 and 0.0695, respectively) (Figure 4). Focusing on the same mouse groups, we further asked whether AA or VH supplements affected number of pups per litter and/or the male to female ratio. The data pertaining to each group are shown in Table S3. However, we found no significant differences across AA- and VH-supplemented groups.

### 3.3. Exposure to AA Prior to Coitus in Males or during Pregnancy in Females Correlated Positively with Offspring Body and Organ Weight

Comparing body weight of PAA and MAA offspring, we noted that weight gain occurred earlier for the latter compared to the former, i.e., in the F2 compared to F3 offspring generation, respectively (see Figure 3). Given that MAA and PAA supplements were administered for 20 and 10 days, respectively, these data suggested a possible effect of AA dose on offspring body weight that was independent on sex and/or developmental window of exposure. Therefore, we asked whether mg of AA exposure correlated with offspring weight, considering MAA and PAA offspring either as separate or as a single experimental group (i.e., PAA+MAA). In either case, milligrams of AA exposure across F1, F2, and F3 offspring generations was calculated in consideration of whether exposure was strictly germline and/or somatic in origin (Figure 2). Identical calculations performed of offspring exposure to VH are shown in Table S2. Across generations, milligrams of germline and/or somatic AA exposure correlated positively with PAA, MAA, and PAA+MAA offspring body weight, with similar tendencies across female and male mice (Table 1). In contrast, correlations between milligrams of VH exposure and body weight were limited to PVH offspring. 

In addition to body weight, we also probed for correlations between milligrams of AA or VH exposure and liver, brain, intestine, testes, and uterus or ovary weight from an average of 10 mice per generation of MAA, PAA, and PAA+MAA offspring groups. Organ weights of each group and their correlations with milligrams of FA supplement are shown in Table S4A,B, respectively. While AA offspring groups showed positive correlations with several organ weights, VH offspring groups only showed few such correlations. This suggested that changes in wet weight of several organs, possibly in addition to body fat, contributed to the increase in body weight of AA offspring. With respect to correlations between milligrams of AA exposure and reproductive parameter-supplemented dams, we found no significant correlation between the number of pups per litter nor male/female offspring ratio (*r* = 0.003 and −0.480, respectively); the corresponding correlation coefficients for VH-supplemented dams were *r* = 0.126 and 0.074, respectively.

### 3.4. Effects of AA Supplementation on Liver FA and DNAm Profiles

The positive correlation between milligrams of AA exposure and offspring liver weight prompted us to ask how AA exposure affected offspring liver FA content. The latter interest stemmed from data showing that THP-1 monocytes stimulated with AA acutely activate the de novo pathway of FA biosynthesis leading to the incorporation of primarily palmitic acid (PA) in neutral lipids and an increase in lipid droplet formation enriched in cis-7-hexadecenoic acid (HDA, 16:1n-9), a positional isomer of palmitoleic acid [34,35]. Specifically, we analyzed liver tissue pooled from three randomly chosen male and female offspring of each supplemented generation with respect to weight and total fat content, in addition to the percent normalized content of four SFA, five MUFA, and seven PUFA (Table S5A,B, respectively). Subsequently, we probed for correlations between these parameters and milligrams of offspring AA exposure across PAA, MAA, and PAA+MAA groups (Table 2). FA were analyzed either by degree of saturation or individually contribution. 

**Figure 5:**
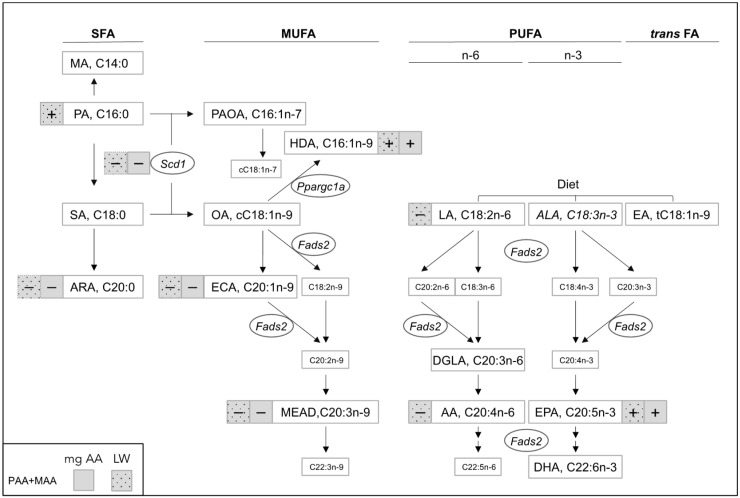
Correlations between milligrams of parental AA exposure or liver weight and offspring liver FA content. Diagram of SFA, MUFA, and PUFA biosynthesis pathways highlighting individual FA that showed significant (*p* < 0.05) or borderline significant (*p* < 0.1) correlations with both germline and germline + somatic PAA+MAA exposure (Pearson’s r). Large white boxes indicate the analyzed FA; FA in italics were not detectable. Squares with solid and dotted filling indicate significant and borderline significant correlations with AA exposure (grey) or liver weight (grey with black dots); “+” and “−“ indicate positive and negative correlations, respectively; promoter methylation was analyzed on the genes indicated in ovals; other abbreviations: SFA, MUFA, and PUFA, saturated, mono-, and polyunsaturated FA, respectively; MA, myristic acid; PA, palmitic acid; SA, stearic acid; ARA, arachidic acid; PAOA, palmitoleic acid; OA, oleic acid; ECA, eicosanoic acid; HDA, cis-7-hexadecenoic acid; LA, linoleic acid; ALA, alpha-linolenic acid; EA, elaidic acid; AA, arachidonic acid; EPA, eicosapentaenoic acid; DHA, docosahexaenoic acid; DGLA, dihomo-γ-linolenic acid; Fads2, fatty acid desaturase 2; Scd1, stearoyl-CoA desaturase 1.

As expected, milligrams of AA exposure correlated positively with liver weight of the randomly chosen pooled samples. Furthermore, several individual FA—rather than FA grouped by degree of saturation—correlated positively or negatively with milligrams of AA exposure. Conversely, milligrams of VH exposure only showed few such correlations. Importantly, most of the significant correlations with AA exposure showed similar tendencies across PAA, MAA, and PAA+MAA offspring groups, with the latter group considering either germline or both germline and somatic exposure (Table 2). Figure 5 illustrates these significantly associated FA in the context of the major pathways of SFA, MUFA, and PUFA synthesis. Notably, two FA with known anti-inflammatory properties (i.e., HDA and eicosapentaenoic acid (EPA)) [35,46] showed positive correlations with milligrams of AA exposure, while the remaining correlations were all negative. The latter included the SFA arachidic acid, in addition to specific n-9 (C20:1n-9, C20:3n-9) MUFA. Notably, all these FA also correlated with liver weight, that additionally correlated positively and negatively with PA and AA, respectively (Table S6, Figure 5). Liver FA data also allowed for the assessment of the influence of dietary linoleic acid (LA), the main precursor of AA. LA of either chow or VH origin (Table S1) is likely to affect the liver AA pool, in addition to exogenous AA exposure. Yet, the significant correlation between hepatic AA and weight was observed in AA-exposed but not VH-exposed mice. If anything, the trends of hepatic AA in AA- and VH-exposed mice suggests that AA metabolism is accelerated in the former group (Table S5). 

Furthermore, we found no significant differences in AA content between AA and VH offspring when supplemented maternally or paternally (*p* > 0.19 in all cases). These results point to the conclusion that dietary LA does not exert any overt effect in our model.

AA-induced HDA synthesis is dependent on SCD1 activity and beta-oxidation, and inhibition of the latter with etomoxir abolishes the AA-induced increase global DNAm in THP-1 cultured monocytes [28,34,35]. Furthermore, exposure to specific FA during lactation has previously been shown to increase global DNAm in adipose tissue of three-month-old mice offspring [22]. Therefore, we asked whether AA supplementation affected offspring global or promoter methylation of *Scd1.* We also surveyed *Ppargc1a* promoter methylation, given its role as important co-regulator of beta-oxidation [47]. We also analyzed *Fads2* promoter methylation since the synthesis of several n-9, n-6, and n-3 FA are dependent on this enzyme. The selected promoter regions analyzed partially overlapped, or were in the vicinity of, DNA regions that had previously been shown to be negatively associated with expression when methylated [48,49]. Importantly, both gene-specific promoter and global DNAm was performed on the same DNA pools analyzed with respect to liver FA. Across offspring generations, *Scd1* and *Ppargc1a* promoter methylation ranged from 2.5–10.9% to 13.8–19.78%, respectively (Table S7), while *Fads2* promoter was completely unmethylated (results not shown). Although liver promoter DNAm of either gene did not differ across one-month-old AA and VH offspring groups, we did uncover significant positive correlations between milligrams of offspring AA exposure and global or *Scd1* promoter methylation in PAA+MAA offspring (Table 3). The latter two also correlated with liver weight in PAA+MAA offspring (Table 3). Furthermore, milligrams of AA and VH showed a positive and negative borderline correlation, respectively, with LW in PAA and MVH progeny. Likewise, LW showed a negative borderline correlation to *Ppargc1a* promoter methylation in MVH offspring, while MAA offspring showed the opposite tendency.

**Figure 6:**
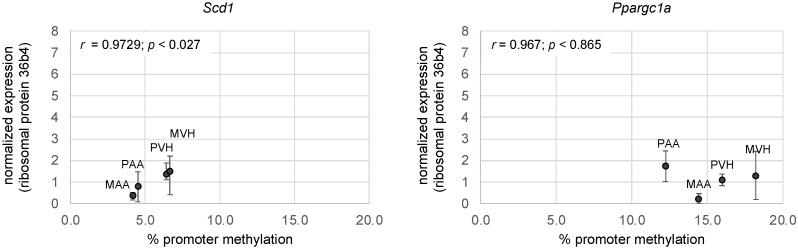
Correlations between Scd1 and Ppargc1a promoter methylation and expression of F2 offspring of AA- and VH-exposed mice. Each data point represents average normalized expression values of *n* = 6 mice (3 male and 3 females); Pearson’s *r* values are indicated in the upper left corner of each graph. Other abbreviations are as in Figure 3.

Finally, we asked whether AA-associated changes in liver *Scd1* or *Ppargc1a* promoter methylation affected gene expression. To that end, we performed RT-PCR on liver samples pooled from 3 male and 3 female offspring (Figure 6). We uncovered a positive correlation between *Scd1* methylation and expression, but not of *Ppargc1a.*

## 4. Discussion

In the present study, BALB/c inbred mice were supplemented for three subsequent generations with a daily dose of AA (0.45 and 0.05% of total FA and energy, respectively) that is close to the AA content in breast milk worldwide [36] and average AA intake in developed countries [50]. A 10 day and 20 day AA supplement, either prior to coitus in male mice or during pregnancy in females, respectively, nearly doubled the body weight of F3 relative to F1 progeny, despite a marginal change in total calories ingested. This is a remarkable response, given that BALB/c mice are relatively resistant to treatments aimed at increasing body weight, such as obesogenic diets [51,52]. The growth-promoting effect of AA has been previously documented. Mice with genetic depletion of endogenous AA due to *Fads2* inactivation respond to AA supplementation by increasing body weight [43,44,45]. In humans, infant plasma AA correlates positively with body weight and growth [53,54], while AA intake is inversely related to stunting in developing countries [40]. However, AA supplementation during pregnancy and/or lactation in mice, rats, or pigs shows either positive or no effects on weight gain [45,55,56,57,58,59,60]. Such discrepancies may reflect differences in the composition of the diet supplied together with AA; the concentration or length of the dietary intervention; or, as our results indicate, prior exposure to AA.

One immediate question is whether our model mimics obesity and obesity-associated adverse traits such as inflammation. The issue is particularly relevant in the light of the altered transport and bioavailability of FA in human obesity, wherein n-6 PUFA are increased [61]. Quantitatively, AA-induced body weight gain was similar to that observed in mice continuously exposed to a LA-enriched and AA-devoid high-fat diet (HFD) for four generations, notwithstanding a roughly threefold higher total fat content of the HFD relative to our supplementation (15.5 vs. 5.1%, respectively) [62]. Likewise, exposure to an LA-enriched HFD (15.5% of total fat from soybean oil) for 16 weeks starting from weaning in mice led to a relative increase in body weight gain akin to that observed in our model [63]. Nonetheless, it cannot be concluded that the response to AA is obesogenic in the present study, as the AA-induced increase in liver weight was not accompanied by an increase in liver total fat content. Conversely, in the two studies just mentioned, exposure to an LA-enriched HFD across or within a generation led to an increase in epididymal or liver fat, respectively. Moreover, we observed an increase in weight of several of the examined organs in response to AA. The data therefore suggest that the increase in body weight cannot be completely explained by adipose tissue expansion.

As for liver FA, a shift towards an anti-inflammatory profile was a distinct feature. AA induced a reduction in LA and AA and an increase in EPA, which were opposite to corresponding profiles observed of mice exposed to the aforementioned LA-enriched HFD. These FA profiles were expected, given that LA and α-linolenic acid (ALA) compete for the same enzyme, FADS2, in the synthesis of AA and EPA, respectively [64]. Interestingly, liver weight of VH offspring only showed a positive correlation with DHA without a concomitant decrease in AA, indicating a possible decoupling of the FADS2 pathway from n-6 PUFA synthesis. The only source of ALA was the Laboratory Rodent diet 5001, suggesting that the accumulation of EPA or DHA is indeed specific to AA or VH exposure, respectively. Furthermore, AA exposure led to an increased levels of the n-9 FA HDA, that akin to EPA has significant anti-inflammatory properties [35,65]. However, while HDA specifically accumulates in lipid droplets following stimulation with AA, EPA is preferentially located in cell membranes, suggesting differential bioavailability or activity [35]. One interesting possibility is that these cellular memories of prior AA exposure function to resolve an inflammatory insult more rapidly by circumventing the need for allocating cellular energy to de novo synthesis of anti-inflammatory FA. These findings suggest that the exposure to AA may exert overall beneficial effects on the innate immune system, which are cumulative across three generations. Whether the known pro- and anti-inflammatory products of AA metabolism contribute to the observed response to AA exposure in addition to the afore mentioned anti-inflammatory FA cannot be concluded from our data.

Our study may have implications for infant nutrition. A 20 day in utero or a 10 day exposure to AA in three-month-old sires and dams for three generations led to a cumulative increase in offspring body weight. Specifically, this suggests that mice retain a memory of AA exposure for at least 84 or 104 days following a 20 day in utero or a 10 day paternal exposure, respectively. Considering that 1 day in a mouse life is the equivalent of ≈40 days of human life [66], our protocol translates to an 1.1 or 2.2 year dietary intervention period in males or females, respectively, that continues to impact on cellular metabolism for at least 9.2 and 11.4 years, respectively. This timeframe is relevant with respect to postnatal formula or breastfeeding in infants and underlines the importance of the active debate concerning whether AA should be included in infant formula products [67,68].

A significant body of independent evidence supports the notion that the observed responses to AA supplementation are specific. Akin to our results, exposure of THP-1 cells to AA also lead to a significant increase in PA, oleic acid, and HDA [34,35]. The remarkable consistencies of AA-induced FA profiles of in vitro or in vivo models suggests that these effects are AA-specific. Furthermore, we can rule out that products of AA oxidation play a significant role in the observed responses on the basis of the following evidence: (1) HDA- and PA-enriched lipid droplet formation in vitro results from exposure to AA, but not to its oxidized metabolites [34,69]; (2) AA oxidation was minimized by storing AA aliquots in soybean oil that contains the antioxidant tert-butylhydroquinone and by limiting exposure to light.

Our data shed light on the effects of lipids on the DNA methylome. HFD trials in adult mice or rats are associated with dynamic, dose-dependent, and/or reversible tissue-specific changes in global DNAm [70,71]. Similarly, short term HFD overfeeding in humans leads to subtle genome-wide changes in DNAm that are only partially reversed after 6-8 weeks [72]. However, two recent studies that investigated whether the mouse liver retains transcriptional, chromatin-structural, or epigenetic memories of HFD-induced obesity following weight loss in mouse led to opposite conclusions [73,74]. The authors argued that the contrasting results in part reflected differences in FA content of the HFD. This would be in accordance with our observation and other studies showing that FA differ in their ability to induce both short- and long-term changes in DNAm [22,28,29] and underlines the importance of homogenizing HFD and control diets for individual FA content [75]. Regarding the potential mechanism of the AA-induced increase in global DNAm, stimulating THP-1 cells with AA leads to an increase in DNAm that is dependent on PPARα activity and beta-oxidation [28]. Interestingly, PPARα is an important regulator of *Scd1* [76], which has recently been shown to be a major regulator of global DNAm in pancreatic β-cells [77]. In accordance, we found that increased exposure to AA was negatively associated with *Scd1* promoter methylation. Taken together, our data point to the importance of PPAR signaling in the hepatic response to AA exposure. Similar cellular responses are likely to occur in the adipose tissue. Reduced liver *Scd1* methylation has previously been shown in C57BL/6 J mice following exposure to an HFD compared to a high-carbohydrate diet. However, while that study found a negative association between promoter *Scd1* methylation and expression [49], that association was positive in this study. Interestingly, exposure to HFD during suckling in rats also leads to epidydimal fat *Scd1* promoter demethylation at weaning that is maintained in adult rats [78]. However, akin to our results in liver tissue, the association between promoter DNAm and expression was positive in young mice, while that association was negative in adult mice. These data suggest that loss of *Scd1* promoter methylation is an early programming event that affects weight gain later in development, in addition to underlining the well-known complex association between DNAm and gene transcription.

Finally, the molecular mechanisms underlying the transfer of information of previous AA exposure across generations are a fascinating topic that can only be speculated. As specific alleles favoring generation-to-generation trait transfer can be discarded as information vehicle in our model, mechanisms must be non-genetic. Epigenetic inheritance has been demonstrated in plants and animal models, but remains controversial [79]. In particular, erasure of most of the epigenetic information during reprogramming in the embryo is difficult to reconciliate with a direct transfer of information contained in DNAm or histones [80]. It has been proposed that to overcome epigenetic erasure, the embryo must activate secondary non-genetic signal that reconstruct the information lost during reprogramming [81]. Proposed reconstructive signals include RNA, DNA-binding proteins, or metabolites. Such mechanisms potentially underlie the cumulative effects of AA in our model. Alternatively, lipids may be proposed as vehicle of information across generations. Lipid droplets embedded in the adipose tissue or elsewhere may be the source of lipid reconstructive signals such as HDA that may be carried by the sperm or established in utero. Communication between adipose and target tissue has been documented for the perivascular adipose tissue, which can transmit proinflammatory signals to the vascular wall and promote atherosclerosis [82].

In conclusion, the data show that the cumulative weight gain phenotype induced by paternal or maternal exposure to AA is distinct from that described of HFD (e.g., LA-rich diets), both with respect the accumulation of n-6 and n-3 FA, and the lack of an increase in liver fat. That, in combination with the cumulative increase the anti-inflammatory FA HDA and EPA suggests that inclusion of AA in infant formula may induce beneficial, heritable effects on the innate immune system. This may be of particular relevance for infants in low-income countries where AA intakes are generally low [40]. However, further studies are necessary to reach incontrovertible evidence and ethically acceptable public health recommendations.

## Figures and Tables

**Table 1:** Correlation between offspring body weight and AA or VH exposure across generations.

Offspring	Tissue Exposed	All	Male	Female
PAA	germline	**0.822** *	0.797	0.917
MAA	germline + somatic	**0.841** *	0.888	0.807
PAA+MAA	germline	**0.720** **	*0.757*	0.704
	germline + somatic	**0.850** ***	**0.867** *	**0.860** *
PVH	germline	**0.845** *	0.736	**0.999** *
MVH	germline + somatic	−0.218	−0.399	0.095
PVH+MVH	germline	0.063	−0.146	0.351
	germline + somatic	0.271	0.080	0.566

In bold, significant correlations (Pearson’s test); * *p* < 0.05; ** *p* < 0.01; *** *p* < 0.001; in italics: *p* < 0.1. MAA, MVH: maternal exposure to AA and VH supplements, respectively; PAA, PVH: paternal exposure to AA and VH supplements, respectively.

**Table 2:** Correlations between FA dose, total fat content, and % FA by degree of saturation and individual FA.

		PAA	MAA	PAA+MAA		PVH	MVH	PVH+MVH	
		gl	gl+s	gl	gl+s	gl	gl+s	gl	gl+s
LW		**0.924** *	**0.825** *	**0.751** **	**0.852** ***	**0.816** *	0.264	0.415	0.492
Total fat		0.601	−0.549	−0.180	−0.205	−0.122	−0.091	−0.098	−0.018
SFA		0.182	0.599	0.168	*0.557*	0.019	−0.084	−0.057	−0.322
MUFA		0.060	0.081	−0.056	0.363	−0.227	−0.690	−0.473	−0.114
PUFA	all	−0.149	−0.467	−0.058	−*0.551*	0.315	0.346	0.333	0.355
	n−3	0.193	0.266	0.263	−0.215	0.609	0.427	0.444	0.547
	n−6	−0.241	−0.535	−0.115	−**0.605** *	−0.173	0.343	0.281	0.225
	n−9	0.103	−0.065	−0.080	0.283	−0.237	−0.699	−0.486	−0.110
Myristic	C14:0	−0.279	−0.140	−0.237	0.170	−0.345	−**0.873** ***	−0.387	−*0.518*
Palmitic	C16:0	0.604	0.350	0.269	**0.579** *	−0.011	−0.103	−0.062	−0.289
Stearic	C18:0	−0.544	0.0332	−0.123	−0.076	0.284	0.010	0.043	−0.105
Arachidic	C20:0	−**0.985** ***	−0.650	−**0.758** **	−*0.498*	−0.341	−0.319	−0.24	−0.235
Palmitoleic	C16:1n−7	−0.546	−0.008	−0.298	0.135	−0.215	−0.68	−0.486	−0.298
HDA	C16:1n−9	0.556	0.586	*0.498*	**0.605** *	−0.099	−0.475	−0.193	0.223
Oleic	cC18:1n−9	0.503	0.118	0.129	0.408	0.0349	−0.636	−0.341	0.051
Elaidic	tC18:1n−9	−0.21	−0.074	−0.163	0.065	−*0.774*	−0.609	−**0.641** *	−0.440
Eicosenoic	C20:1n−9	−0.723	−*0.803*	−*0.547*	−**0.637** *	−0.471	−0.499	−0.431	−0.312
MEAD	C20:3n−9	−0.693	−*0.751*	−**0.741** **	−*0.544*	−0.454	−0.601	−*0.517*	−0.148
Linoleic	C18:2n−6	−0.342	−**0.268**	−0.027	−*0.549*	−0.326	0.226	0.131	0.0826
Arachidonic	C20:4n−6	−0.071	−**0.870** *	−0.260	−**0.592** *	0.179	0.454	0.412	0.316
DGLA	C20:3n−6	−0.294	0.186	0.081	−0.345	0.034	0.246	0.122	0.192
EPA	C20:5n−3	*0.736*	**0.852** *	**0.766** **	**0.781** **	0.026	0.205	0.161	0.114
DHA	C22:6n−3	0.116	0.061	0.174	−0.317	0.642	0.486	0.484	**0.604** *

Correlations were performed on percent normalized FA; in bold, significant correlations as determined by Pearson’s test; * *p* < 0.05; ** *p* < 0.01; *** *p* < 0.001; in italics, *p* < 0.1; gl and gl+s (germline and germline + somatic, respectively) refer to offspring exposure to AA or VH; LW, liver weight; HDA, cis-hexadecenoic acid; MEAD, eicosatrienoic acid; DGLA, dihomo-gamma-linolenic acid; EPA, eicosapentaenoic acid; DHA, docosahexaenoic acid.

**Table 3:** Correlations between FA dose or liver weight and global and gene-specific DNA methylation.

				Promoter Methylation			
		Global DNAm		*Scd1*		*Ppargc1a*	
Offspring	Tissue Exposed	mg FA	LW	mg FA	LW	mg FA	LW
PAA	germline	0.646	*0.759*	−0.425	−0.632	0.285	0.329
MAA	germline + somatic	0.526	0.669	−0.697	−0.725	−0.467	−0.612
PAA+MAA	germline	0.488	**0.729** **	−**0.643** *	−*0.511*	−0.277	−0.105
	germline + somatic	**0.600** *		−0.398		0.015	
PVH	germline	0.493	0.499	−0.139	−0.036	−0.384	−0.220
MVH	germline + somatic	−0.676	−*0.797*	0.191	0.362	0.326	*0.757*
PVH+MVH	germline	−0.071	0.015	0.120	0.175	0.001	0.172
	germline + somatic	−0.247		0.070		0.169	

In bold, significant correlations as determined by Pearson’s test; * *p* < 0.05; ** *p* < 0.01; in italics, *p* < 0.1. Global DNAm and promoter methylation were determined of liver tissue pooled from three male or female offspring of AA- or VH-supplemented mice. LW: liver weight.

## Data Availability

Not applicable.

## Supplementary Materials

The following supporting information can be downloaded at https://www.mdpi.com/article/10.3390/cells11061057/s1, Figure S1: Growth and food intake of three-month-old founder mice supplemented with AA or VH. Figure S2: Body weight of female and male one-month-old offspring following germline or in utero FA exposure for three consecutive generations. Table S1: Fatty acid profile of the Laboratory Rodent diet 5001 and vehicle (Nutrioli^®^ soybean oil). Table S2: FA supplementation across generations and offspring weight. Table S3: Reproductive parameters of FA-supplemented and non-supplemented three-month-old dams. Table S4: Offspring organ weight and correlation with mg of FA exposure. Table S5: Analysis of liver tissue of one-month old offspring of FA-supplemented mice. Table S6: Correlations between liver weight, total fat, and FA (individual and by degree of saturation) across three offspring generations. Table S7: Global DNAm and *Scd1*. *Ppargc1a* promoter methylation of liver from one-month-old offspring of FA supplemented mice.
