# Supplementary material for: Cumulative Metabolic and Epigenetic Effects of Paternal and/or Maternal Supplementation with Arachidonic Acid across Three Consecutive Generations in Mice
Source: Cells. 2022 Mar 21;11(6):1057. doi: 10.3390/cells11061057 (PMC8947399; doi:10.3390/cells11061057)
Supplement: Supplementary file 1 [file cells-11-01057-s001.zip › cells-1601211-supplementary.pdf]

A. Baseline weight of F0 founder mice.

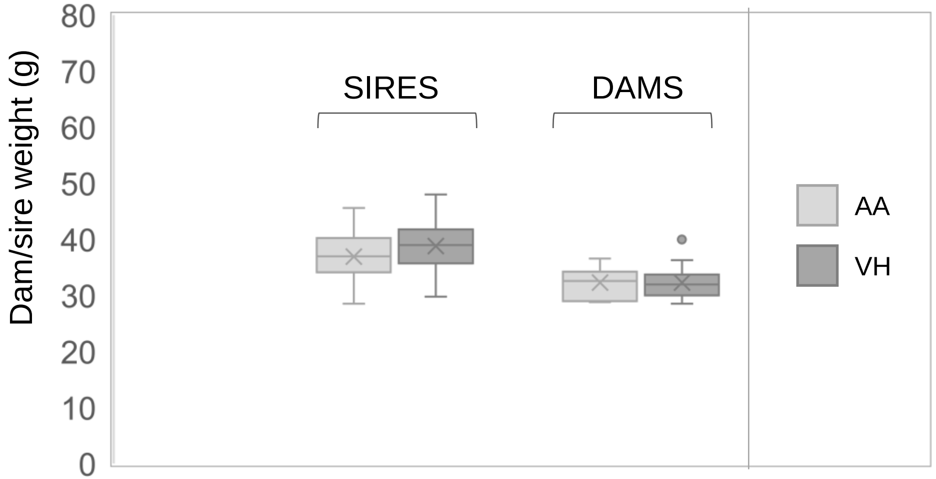

B. Relative weight gain following FA supplementation.

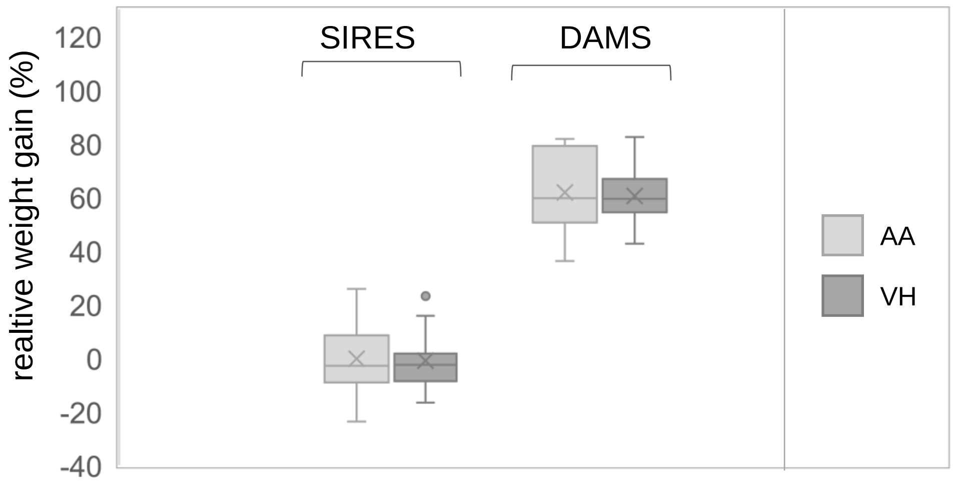

C. Food intake of FA supplemented mice.

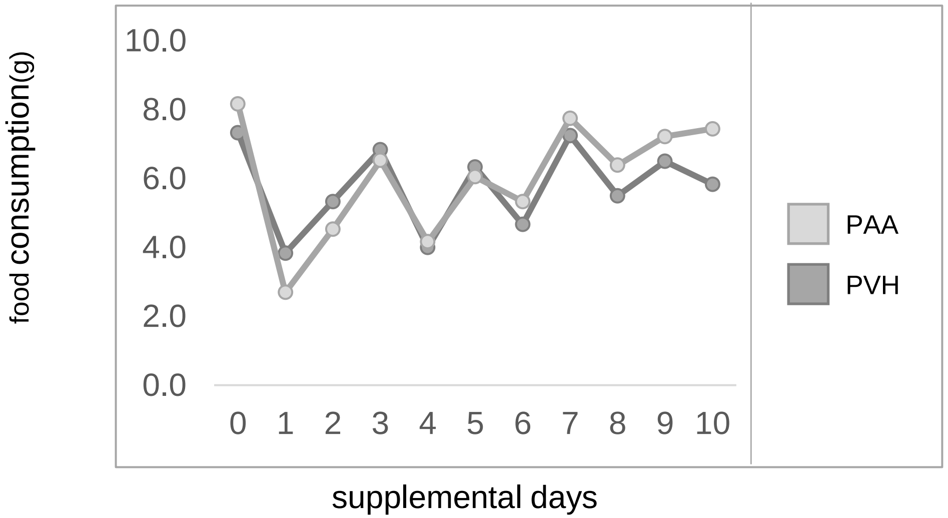

Figure S1. Growth and food intake of three-month-old founder mice supplemented with AA or VH. A) Initial weight of F0 (founder) generation of sires and dams; **B**) Relative weight gain (%) following AA or VH exposure; **C**) Food intake during 10 day supplemental period of sires; of note: food intake was not representative of the founder mice in **A**); in those experiments food intake was not recorded since dams were not housed separately; the data pertaining to food intake represent an identical supplemental experiment carried out in 2020 (n=3 male mice) that showed no effect of a daily AA exposure (1.35 mg) for 10 days on weight (results not shown); AA: arachidonic acid dissolved in vehicle; VH: vehicle only.

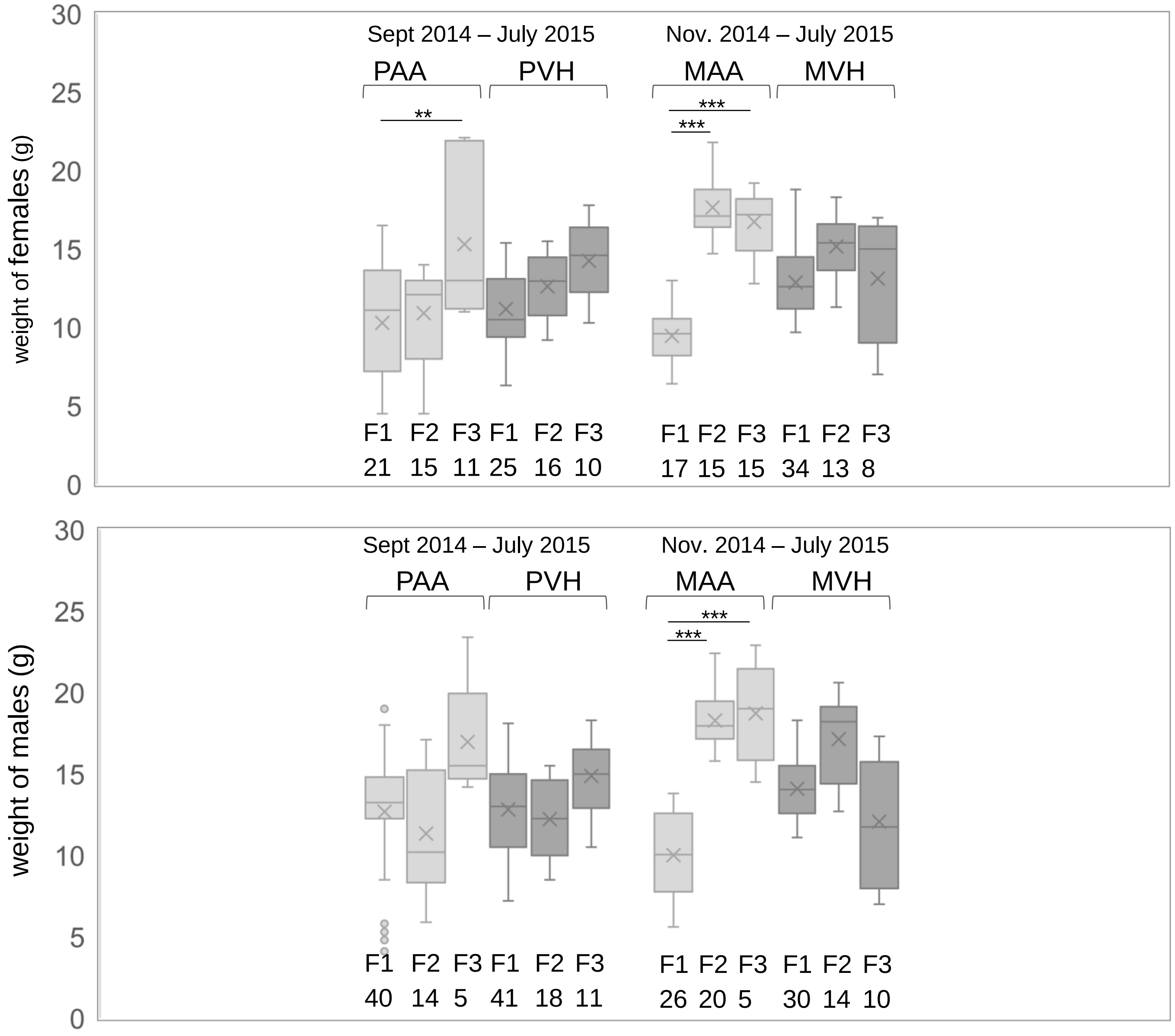

Figure S2. Body weight of female and male one-month-old offspring following germline or in utero AA- or VH-exposure for three consecutive generations. Y-axis indicates female and male offspring body weight (upper and lower graphs, respectively). Exposure months and “n” of each group are indicated below each bar, respectively; horizontal lines indicate significant differences across experimental groups; **p<0.01; ***p<0.001; PAA and MAA: paternal and maternal AA supplementation, respectively; PVH and MVH: paternal and maternal VH exposure, respectively.
